# Supplementary material for: Novel Necroptosis-Related Gene Signature for Predicting Early Diagnosis and Prognosis and Immunotherapy of Gastric Cancer
Source: Cancers (Basel). 2022 Aug 11;14(16):3891. doi: 10.3390/cancers14163891 (PMC9405737; doi:10.3390/cancers14163891)
Supplement: Supplementary file 1 [file cancers-14-03891-s001.zip › cancers-1813198-supplementary.pdf]

Table S1. Accuracy of early diagnosis.

	GC	Normal	All	Accuracy
**3-mRNA positive**	26	3	29	89.7
**3-mRNA negative**	2	10	12	83.3
**All accuracy**				87.8

NA: not available.

A

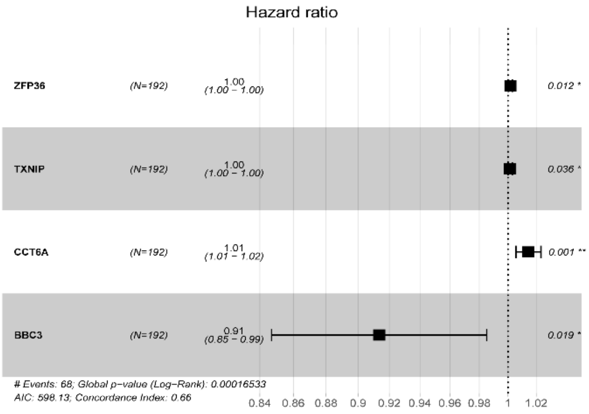

B

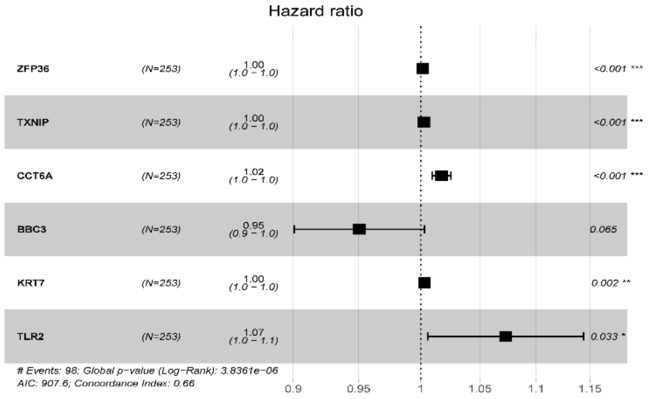

C

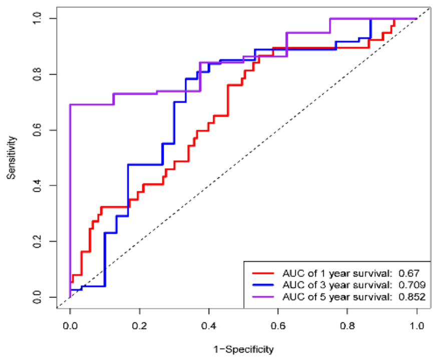

D

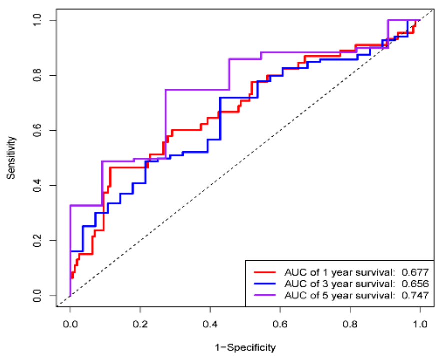

Figure S1. Prognostic risk score construction of other proportions divide the TCGA dataset. (**A**) Results of multivariate cox proportional risk regression analysis of TCGA dataset according to 5:5. (**B**) Results of the multivariate cox proportional risk regression analysis of the TCGA data set by 2:1. (**C**) The ROC identification of TCGA data set according to 5:5. (**D**) The ROC identification of the TCGA data set according to 2:1.
